# Single-Cell Transcriptome Data Clustering via Multinomial Modeling and Adaptive Fuzzy K-Means Algorithm

**DOI:** 10.3389/fgene.2020.00295

**Published:** 2020-04-17

**Authors:** Liang Chen, Weinan Wang, Yuyao Zhai, Minghua Deng

**Affiliations:** ^1^School of Mathematical Sciences, Peking University, Beijing, China; ^2^Mathematical and Statistical Institute, Northeast Normal University, Changchun, China; ^3^Center for Quantitative Biology, Academy for Advanced Interdisciplinary Studies, Peking University, Beijing, China

**Keywords:** single-cell RNA sequencing, UMI count data, deep autoencoder, statistical modeling, adaptive fuzzy k-means clustering

## Abstract

Single-cell RNA sequencing technologies have enabled us to study tissue heterogeneity at cellular resolution. Fast-developing sequencing platforms like droplet-based sequencing make it feasible to parallel process thousands of single cells effectively. Although a unique molecular identifier (UMI) can remove bias from amplification noise to a certain extent, clustering for such sparse and high-dimensional large-scale discrete data remains intractable and challenging. Most existing deep learning-based clustering methods utilize the mean square error or negative binomial distribution with or without zero inflation to denoise single-cell UMI count data, which may underfit or overfit the gene expression profiles. In addition, neglecting the molecule sampling mechanism and extracting representation by simple linear dimension reduction with a hard clustering algorithm may distort data structure and lead to spurious analytical results. In this paper, we combined the deep autoencoder technique with statistical modeling and developed a novel and effective clustering method, scDMFK, for single-cell transcriptome UMI count data. ScDMFK utilizes multinomial distribution to characterize data structure and draw support from neural network to facilitate model parameter estimation. In the learned low-dimensional latent space, we proposed an adaptive fuzzy k-means algorithm with entropy regularization to perform soft clustering. Various simulation scenarios and the analysis of 10 real datasets have shown that scDMFK outperforms other state-of-the-art methods with respect to data modeling and clustering algorithms. Besides, scDMFK has excellent scalability for large-scale single-cell datasets.

## 1. Introduction

In the past decade, high-throughput sequencing technology has been widely used in various fields of biology and medicine, greatly promoting research in related areas (Reuter et al., 2015). Transcriptome sequencing can be applied to measure and describe the expression of gene transcription or the cell status of all kinds of species. However, traditional bulk sequencing technology is based on a group of cells, but each sample contains hundreds and thousands of cells. Therefore, the final sequencing data represent the average expression levels of genes in a group of cells, concealing the heterogeneity of gene expression among cells (Rowen et al., 1997). In recent years, however, exciting single-cell transcriptome sequencing technology has been booming, allowing researchers to reveal the expression of all cells in the whole genome at the cellular level, in turn facilitating cell heterogeneity and tissue differentiation research (Shapiro et al., 2013; Patel et al., 2014; Kolodziejczyk et al., 2015; Wang and Navin, 2015).

Early single-cell sequencing technologies like Smart-seq2 or MATQ-seq can measure the full length of transcripts, but have small cell throughput and are somewhat expensive (Picelli et al., 2013; Sheng et al., 2017). Recently developed droplet-based sequencing technologies, such as 10x Chromium and Drop-seq, can efficiently profile a large number of cells in parallel with high throughput in a single experiment (Svensson et al., 2017; Zheng et al., 2017). High efficiency and low consumption make it possible for such technologies to bring revolutionary insight, and they have thus gained popularity. In addition, droplet-based sequencing technologies utilize a Unique Molecular Identifier (UMI) to annotate the 3'-end of the transcript, resulting in the reduction of amplification bias of polymerase chain reaction(PCR) (Islam et al., 2014). Therefore, in this article, we have focused on the research and analysis of single-cell RNA-seq UMI count data.

Proper execution of scRNA-seq data analysis requires the identification and characterization of cell subtypes (Macosko et al., 2015), which contributes to the appraisal of differentially expressed genes (Kharchenko et al., 2014; Finak et al., 2015) and construction of a gene expression regulation network (Aibar et al., 2017; Fiers et al., 2018). However, an inefficient RNA capture procedure leads to a failure to detect low-expressed genes, defined as a dropout event, resulting in substantial zero counts in data (Grün et al., 2014; Hebenstreit, 2018). Thus, clustering for extremely noisy, high-dimensional, and massive single-cell transcription profiles poses a severe challenge to researchers (Kiselev et al., 2019). Most existing clustering algorithms customized for single-cell analysis do not model and denoise such data. Typically, they first learn the predefined distance measure and similarity metric based on an original data matrix directly or a reduced data matrix by simple linear dimension reduction methods, like PCA and ICA, and then utilize the traditional hard clustering methods, such as standard k-means clustering (Marco et al., 2014; Grün et al., 2016), graph-based spectral clustering (Wang et al., 2017; Zhu et al., 2019) or community detection (Levine et al., 2015; Satija et al., 2015), density-based clustering (Jiang et al., 2016), integrated learning clustering (Kiselev et al., 2017; Yang et al., 2018), and hierarchical clustering (Zeisel et al., 2015; Lin et al., 2017). However, in addition to the possibility of spurious identification of cell subtypes by separating dimension reduction and clustering, expensive computation limits their performance on large-scale datasets.

Understanding the generating mechanism of UMI count data is essential to developing model-based statistical methods for single-cell transcriptome analysis. Log-normal, poisson, negative binomial distributions are several mainstream modeling distributions in studying single-cell transcriptome profiles (Pierson and Yau, 2015; Risso et al., 2018). To account for redundant zero counts, researchers are motivated to design mixture version distributions with a zero-inflation component, such as zero-inflated lognormal, zero-inflated poisson, and zero-inflated negative binomial models. Recently, however, studies have shown that single-cell UMI count data follow multinomial distribution without zero-inflation and that current normalization procedure distort UMI count data (Townes et al., 2019). Sun et al. (2017) proposed a dirichlet multinomial bayesian mixture model named DIMM-SC for droplet-based single-cell transcriptomic data clustering. However, DIMM-SC requires a sophisticated numerical algorithm to reduce the high computational cost, and it ignores the measurement errors and uncertainties buried in the UMI count data.

Deep learning technology has shown amazing capabilities in unsupervised representation learning, which can efficiently learn potentially vital non-linear features in data (Eraslan et al., 2019a; Zou et al., 2019). An autoencoder is a commonly used neural network structure composed of an encoder and a decoder, which is specifically designed for feature extraction and dimension reduction of high-dimensional data (Hinton and Salakhutdinov, 2006). The encoder is responsible for compressing and mapping the input vector to obtain low-dimensional representation, and the decoder maps this representation back to the high-dimensional space to gain a reconstructed vector. Thanks to the non-linear gating unit function, autoencoders can learn the underlying low-dimensional manifold structure of high-dimensional data and effectively capture non-linear complex dependencies among samples and features. According to variety of reconstruction loss functions and latent space assumptions, autoencoders can be divided into different categories. Amodio et al. (2019) proposed a deep multitasking neural network model called SAUCIE and utilized the standard mean square error as data denoising loss function, which may underfit the single cell RNA-seq data and is unable to recover the cell types (Eraslan et al., 2019b). Deng et al. (2019) proposed an iterative deep recurrent learning model called scScope to simultaneously realize the imputation and clustering for single-cell transcriptome data. ScScope utilized the mask mean square error as the reconstruction loss function, but it did not explicitly aggregate the clustering function into the training process. Arisdakessian et al. (2019) proposed the DeepImpute model for fast imputation of single-cell RNA-seq data. Its reconstruction loss function was a weighted mean square error objective function. Similarly, DeepImpute did not consider adding clustering learning procedure in latent space. Eraslan et al. (2019b) proposed a denoising autoencoder model called DCA for single-cell counts data, which assumed that single-cell UMI count data followed negative binomial or zero-inflated negative binomial distribution. On the basis of DCA, Tian et al. (2019) proposed scDeepCluster and used a self-trained objective function to achieve the clustering target in the learned low-dimensional latent space. However, they did not consider pre-selecting informative genes as inputs, resulting in high requirements of memory and running time. Moreover, Hafemeister and Satija (2019) have recently pointed out that an unconstrained negative binomial model may overfit the scRNA-seq data by the constraints of genes with similar abundances. Grønbech et al. (2018) proposed a variational autoencoder model called scVAE for single-cell count data clustering. ScVAE presupposed that the feature distribution of the latent space followed Gaussian or mixture Gaussian distribution, and then applied variational inference to derive a learnable ELBO objective function. However, the Gaussian or mixture Gaussian assumption may put too many constraints on the latent space. Besides, variational inference has high requirements for the optimization technique.

In this paper, we combined statistical modeling and deep learning techniques to learn a more appropriate latent space representation suitable for clustering. We have proposed a model called scDMFK that simultaneously performs data denoising, dimensionality reduction, and clustering. We first utilized multinomial distribution to characterize single-cell UMI count data, where the proportional parameters of multinomial distribution are learned using deep autoencoder. In latent space, we have proposed a fuzzy weighted k-means clustering algorithm with adaptive loss function and entropy regularization. The cluster membership assignment probability of cells can be derived by soft assignment criterion explicitly in closed form instead of being updated by a stochastic gradient descent of the neural network back-propagation algorithm. Different simulation scenarios and several real dataset results show that our model is superior to other benchmarked methods for scRNA-seq data clustering. Moreover, modeling UMI count data based on multinomial distribution is more effective than the commonly used negative binomial distribution and non-parametric mean square error with respect to cell type identification.

## 2. Methods

### 2.1. Multinomial Modeling for Single-Cell UMI Count Data

We begin with some notations. Suppose single-cell UMI count data matrix is *X*_*ij*_(1 ≤ *i* ≤ *n*, 1 ≤ *j* ≤ *m*), *n* is cell number, and *m* is the feature(gene) number. Then $n_{i}=\overset{m}{\underset{j=1}{\sum }}X_{ij}$ represents the total UMI counts in the *i*-th cell. Assume that the *i*-th cell contains *t*_*i*_ total mRNA transcripts and that *Y*_*ij*_(1 ≤ *i* ≤ *n*, 1 ≤ *j* ≤ *m*) is the underlying mRNA transcripts matrix. Then, when we process and lyse the *i*-th cell on the sequencing protocol, *t*_*i*_ mRNA transcripts are attached by barcodes and UMIs such that they are transformed to cDNA molecules by reverse transcription. After removing PCR duplicates, we generate the *n*_*i*_ UMI counts in the *i*-th cell. Because reverse transcriptase is an inefficient and error-prone enzyme, causing some fraction of the cDNA molecules to be lost, *n*_*i*_ is usually much less than *t*_*i*_. Thus, the process of successfully converting mRNA to UMI is actually a random sampling process. We defined *p*_*ij*_ as the relative abundance of the amount of mRNA expressed by *j*-th gene shared in total mRNA of *i*-th cell, namely,

(1) $$\begin{array}{l} p_{ij}=\frac{y_{ij}}{t_{i}}=\frac{y_{ij}}{\sum_{j=1}^{m}y_{ij}} \end{array}$$

Considering *n*_*i*_ ≪ *t*_*i*_ and true transcripts counts *y*_*ij*_ are unknown, we supposed that UMI counts *X*_*ij*_ are samples of *y*_*ij*_ with relative abundances remaining constant; thus, the probability distribution function of *X*_*i*_ = (*X*_*i*1_, *X*_*i*2_, …, *X*_*im*_) is multinomial distribution with parameter vector *p*_*i*_ = (*p*_*i*1_, *p*_*i*2_, …, *p*_*im*_) to be estimated,

(2) $$\begin{array}{l} f_{i}\left(X_{i}\right)=\frac{n_{i}!}{X_{i1}!X_{i2}!\ldots X_{im}!}\overset{m}{\underset{j=1}{\prod }}p_{ij}^{X_{ij}} \end{array}$$

Because of non-linear dependencies among genes and complex associations between cells, the parameter vectors *p*_*i*_(1 ≤ *i* ≤ *n*) corresponding to each cell are not completely free from statistical theory. However, these dependencies cannot be captured by a simple generalized linear model because unknown parameters *p*_*ij*_(1 ≤ *i* ≤ *n*, 1 ≤ *j* ≤ *m*) actually fall on a low-dimensional manifold. Therefore, instead of designing a specific non-linear association expression or bayesian priors, like mixture dirichlet distribution, we utilized deep autoencoders to approximate the underlying manifold and learn parameter vectors *p*_*i*_(1 ≤ *i* ≤ *n*). Furthermore, owing to frequent dropout events, we could not ignore the impact of this extra noise on parameter estimation. To model the dropout events, we introduced binary random variables *U*_*ij*_, where *U*_*ij*_ = 0 represents that the *j*-th gene drops out in the *i*-th cell. Letting π_*ij*_ = *P*(*U*_*ij*_ = 1), we have

(3) $$\begin{array}{l} U_{ij}\sim Bernoulli\left(\pi_{ij}\right) \end{array}$$

Obviously, low-expressed genes have high probabilities of dropping out, which implies that π_*ij*_ is positively correlated with true expression level of *j*-th gene in *i*-th cell. Assume that *V*_*ij*_ is the expected relative expression level of *j*-th gene in *i*-th cell. Then, given the dropout phenomenon, *p*_*ij*_ should be the element-wise product of π_*ij*_ and *V*_*ij*_ and then normalized to sum to one.

(4) $$\begin{array}{l} p_{ij}=\frac{\pi_{ij}V_{ij}}{\sum_{j=1}^{m}\pi_{ij}V_{ij}} \end{array}$$

In this case, (π_*ij*_, *V*_*ij*_)(1 ≤ *i* ≤ *n*, 1 ≤ *j* ≤ *m*) make up the parameters to be estimated.

Autoencoders are widely utilized to realize data compression coding and data reconstruction coding in representation learning. Suppose the latent space that the encoder maps the input into is *Z*: we adopted denoising autoencoder architecture similar to that of DCA (Eraslan et al., 2019b) and outputted two groups of tensors, one for π and another for *V*. Specifically,

(5) $$\begin{array}{l} Z=Encoder\left(X\right) \end{array}$$

(6) $$\begin{array}{l} \hat{X}=Decoder\left(Z\right) \end{array}$$

(7) $$\begin{array}{l} \pi =sigmoid\left(\hat{X}W_{\pi }\right) \end{array}$$

(8) $$\begin{array}{l} V=\exp\left(\hat{X}W_{V}\right) \end{array}$$

where *W*_π_ and *W*_*V*_ are neural network parameters. We utilized the sigmoid function as the activator of π because the dropout probability ranges from zero to one. In fact, this motivation stems from the observable probability that π_*ij*_ = *P*(*U*_*ij*_ = 1) can be modeled as a logistic regression function of underlying true relative expression level. We selected the exponential function as the activation function of *V* for its non-negativity. To construct the data reconstruction loss function, we assumed that, given latent variable *Z*, the samples *X* are conditionally independent. Therefore, we can naturally took the negative log-likelihood of multinomial distribution as the reconstruction loss, as

(9) $$\begin{array}{l} L_{1}=-\log P_{multinomial}\left(X|\pi ,V,Z\right) \end{array}$$

(10) $$\begin{array}{l} =-\log\overset{n}{\underset{i=1}{\prod }}\frac{n_{i}!}{X_{i1}!X_{i2}!\ldots X_{im}!}\overset{m}{\underset{j=1}{\prod }}p_{ij}^{X_{ij}} \end{array}$$

(11) $$\begin{array}{l} \propto -\overset{n}{\underset{i=1}{\sum }}\overset{m}{\underset{j=1}{\sum }}X_{ij}\log p_{ij} \end{array}$$

where the value of *p*_*ij*_ can be calculated by Equation (4). When the proportion of zero counts in the data is not so high, we also considered estimating *p*_*ij*_ directly; that is to say, we chose the softmax activation function at the last layer of decoder and outputted the estimation of *p*_*ij*_ directly. For distinguishing, we called the standard model scDMFK and the alternative model D-scDMFK.

### 2.2. Fuzzy k-Means Clustering With Adaptive Loss

Instead of performing the cell clustering procedure in denoising reconstruction space, we took full advantage of the low-dimensional latent space *Z* learned by the encoder. The K-means algorithm and its extensions have been the most commonly used clustering methods because of their efficiency. Assuming *K* subpopulations in latent space, we utilized a fuzzy k-means algorithm with adaptive distance measurement and entropy regularization to perform clustering (Zhang et al., 2019). Its optimization objective function can be written as

(12) $$\begin{array}{l} \underset{W,\mu_{j}}{\min}\overset{n}{\underset{i=1}{\sum }}\overset{K}{\underset{j=1}{\sum }}w_{ij}\frac{(1+\sigma )\|z_{i}-\mu_{j}{\|}_{2}^{2}}{{\left\|z_{i}-\mu_{j}\right\|}_{2}+\sigma }+\text{λ}w_{ij}\log w_{ij} \end{array}$$

(13) $$\begin{array}{l} s.t.\overset{K}{\underset{j=1}{\sum }}w_{ij}=1,0<w_{ij}<1,1<i<n. \end{array}$$

where μ_*j*_ is the *j*-th cluster center, and *z*_*i*_ represents the low-dimensional representation of the *i*-th cell. *w*_*ij*_ can be regarded as the probability that the *i*-th cell belongs to the *j*-th cluster. Besides, σ and λ are two non-negative hyperparameters. For convenience, we denoted $\frac{\left(1+\sigma \right){\left\|z_{i}-\mu_{j}\right\|}_{2}^{2}}{{\left\|z_{i}-\mu_{j}\right\|}_{2}+\sigma }$ as ‖*z*_*i*_ − μ_*j*_‖_σ_. When σ → 0, then ‖*z*_*i*_ − μ_*j*_‖_σ_ → ‖*z*_*i*_ − μ_*j*_‖_2_. In turn, when σ → ∞, we have ${\left\|z_{i}-\mu_{j}\right\|}_{\sigma }\to {\left\|z_{i}-\mu_{j}\right\|}_{2}^{2}$. Thus, σ is a trade-off parameter that controls the robustness to various outlier types (see Supplementary Material). The entropy regularization is introduced for avoiding trivial solution, i.e., *w*_*ij*_ = 1 if *z*_*i*_ is assigned to the *j*-th cluster and *w*_*ij*_ = 0, otherwise. This hard assignment procedure makes us update the cluster label to each data point manually, which does not contribute to the efficiency of stochastic gradient descent and may lead to collapse of different clusters. Based on information theory, larger entropy represents higher disorder. So λ is one trade-off parameter that controls the distribution of *w*_*ij*_. Actually, when latent space representation *z*_*i*_(1 ≤ *i* ≤ *n*) and cluster center μ_*j*_(1 ≤ *j* ≤ *K*) are known, *w*_*ij*_ has an explicit close-form solution for above optimization problem, which is

(14) $$\begin{array}{l} w_{ij}=\frac{\exp\left(-\frac{{\left\|z_{i}-\mu_{j}\right\|}_{\sigma }}{\lambda }\right)}{\sum_{l=1}^{K}\exp\left(-\frac{{\left\|z_{i}-\mu_{l}\right\|}_{\sigma }}{\lambda }\right)} \end{array}$$

Therefore, we naturally constructed the following adaptive fuzzy k-means loss function,

(15) $$\begin{array}{l} L_{2}=\overset{n}{\underset{i=1}{\sum }}\overset{K}{\underset{j=1}{\sum }}w_{ij}{\left\|z_{i}-\mu_{j}\right\|}_{\sigma } \end{array}$$

where *w*_*ij*_ is given by Equation (14) and is the adaptive weighted coefficient representing the soft allocation probability of assigning the *i*-th cell to the *j*-th cluster membership. After each iteration of training, we can assign the *i*-th cell to the cluster label corresponding to the largest *w*_*ij*_(1 ≤ *j* ≤ *K*).

### 2.3. Training Objective and Parameter Setting

**Figure 1:**
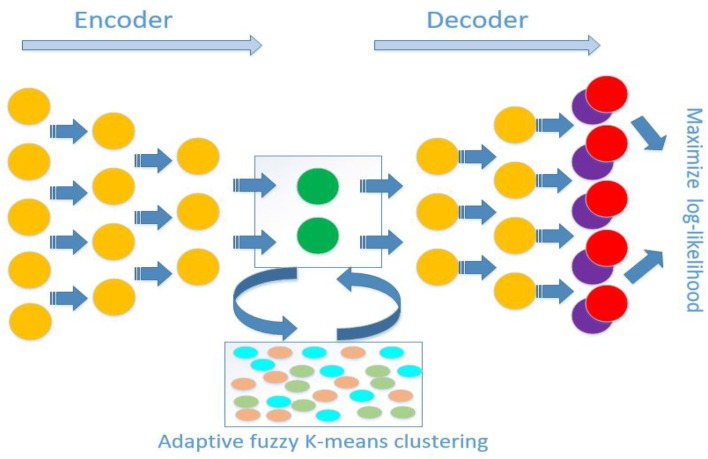
Schematic diagram of antecedent neural network architecture for scDMFK. The encoder and decoder are symmetrical structures, and the outputs are multinomial modeling parameters. In latent space, embedded points are clustered using an adaptive fuzzy k-means clustering algorithm. The overall training objective is to maximize log-likelihood function and minimize adaptive fuzzy k-means clustering loss function simultaneously.

Having finished whole model construction, we summarize the two components: denoising autoencoder based on multinomial modeling and fuzzy soft k-means clustering with adaptive loss. The total training objective function is given as

(16) $$\begin{array}{l} L\left(\pi ,V,Z|X\right)=L_{1}+\alpha L_{2} \end{array}$$

where hyperparameter α controls the relative importance of data generation and data clustering. A simple model schematic is shown in Figure 1. When we input data to the network, it obtains the latent representation *Z* through the encoder and hidden layer, which can calculate the *L*_2_ clustering loss. Then the low-dimensional representation *Z* forwards to the model output through the decoder, which is brought into *L*_1_ loss to calculate the negative log-likelihood. The weight of neural network and cluster centers can be jointly optimized and updated by stochastic gradient descent and a back-propagation algorithm. We implement our model in Python 3 using deep learning software Tensorflow. During the following simulation and real data experiments, we took α as 1 by default. The default values of hyperparameter σ and λ were also 1, and their optimal setting for particular datasets have been discussed in section 3. The optimizer for whole model is Adam with a learning rate 0.0001. For autoencoder network architecture, the sizes of two hidden layers were set to 256 and 64 in the encoder. The decoder is the reverse structure of the encoder, and the bottleneck layer (the latent space) had a size of 32. The minibatch size was set to 256 during the training process. As for the model training strategy, we first pre-trained *L*_1_ loss by 1,000 epochs and then initialize cluster centers μ_*j*_(1 ≤ *j* ≤ *K*) by standard k-means algorithm in the learned latent space. Lastly, we trained the whole *L*(π, *V, Z*|*X*) loss function until cluster membership assignments did not change.

### 2.4. Data Preprocessing

We collected the scRNA-seq UMI count data matrix (cells by genes) after passing quality control. We first discarded the genes that are not expressed in any cell and then filtered the cells without gene expression. Then, we calculated the normalized dispersion value of each gene and pick the top 500 highly variable genes from the original whole genes based on dispersion ranking. The expression of these genes was used as input to the model. Based on the consideration of neural network numerical stability, we transformed discrete count data into continuous smooth data. Specifically, we first normalized the total count amount of each cell to their median value level and then utilized a log-transformation for data. Finally, we transformed the logarithm data into z-score data, which means that each selected gene has zero mean and unit variance. All the above preprocessing procedures can be accomplished by using the scanpy package (Wolf et al., 2018).

## 3. Results

### 3.1. Competing Methods and Evaluation Index

We selected two traditional statistic-based scRNA-seq clustering algorithms, SIMLR (Kiselev et al., 2017) and CIDR (Lin et al., 2017), as competing methods. Considering the high time consumption, we used a close version of SIMLR during large-scale dataset experiments. For deep learning-based methods, we chose recently published scDeepCluster (Tian et al., 2019) as the competitor since it also combines dimensionality reduction and clustering. These three methods were tested with their default procedure and parameter setting in both simulation and real data analysis. In addition, to demonstrate the advantages of multinomial distribution modeling, we compared it with three other existing deep learning based scRNA-seq data denoising models: DCA (Eraslan et al., 2019b), scScope (Deng et al., 2019), and DeepImpute (Arisdakessian et al., 2019). We substituted the zero-inflated negative binomial model, masked MSE, and weighed MSE for the multinomial denoising model, respectively (see Supplementary Material). These three models were combined with our proposed adaptive fuzzy k-means clustering algorithm to organize three competitors and we denoted them as ZINB, mask MSE, and weight MSE, respectively. All competing methods were run on the same computer sever with Ubuntu 16.04. We use two metrics, adjusted Rand index (ARI) and normalized mutual information (NMI), to evaluate the clustering performance of each algorithm. A larger ARI and NMI value dreflect better performance of the cluster algorithm.

### 3.2. Splatter Simulation

**Figure 2:**
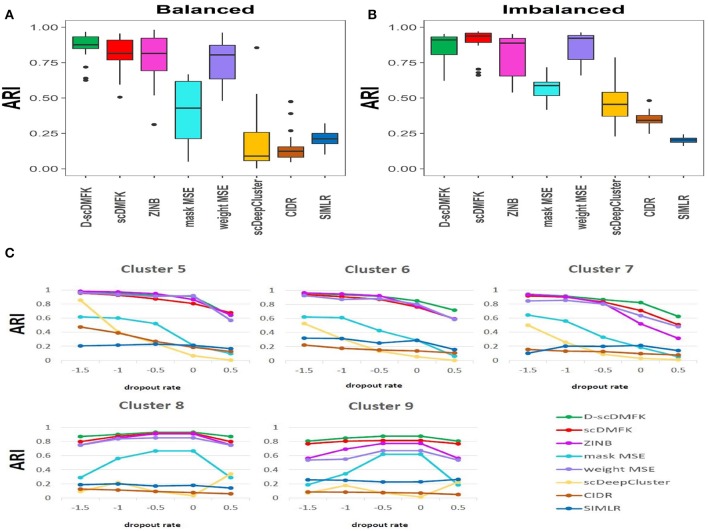
Simulation analysis. **(A,B)** Boxplots of ARI values in Splatter balanced and imbalanced simulation, respectively. **(C)** Change of ARI values with the increasing dropout rate in Splatter balanced experiment.

**Figure 3:**
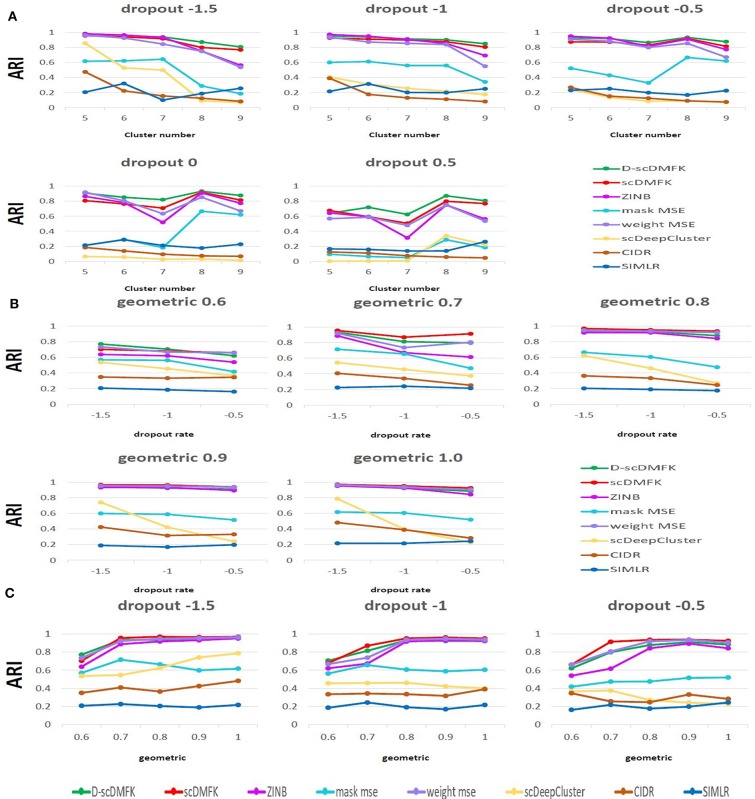
Simulation analysis. **(A)** Change in ARI values with the increasing cluster number in Splatter balanced experiment. **(B,C)** Change in ARI values with the increasing dropout rate and geometric in Splatter imbalanced experiment.

We first used the most popular simulation package, Splatter (Zappia et al., 2017), to generate our simulation data. The core of the Splatter model is a gamma-poisson distribution where mean expression levels for each gene are simulated from a gamma distribution, and the biological coefficient of variation is used to enforce a mean-variance trend before counts are simulated from a poisson distribution. Our simulation experiments were mainly divided into two parts: balanced experiment and imbalanced experiment. The main difference between them was whether the number of cells in each cluster was consistent. In the balanced experiment, the number of cells in each cluster was 500. We explored the performance of each method under different cluster numbers and dropout ratios, where the number of clusters ranged from 5 to 9, and the dropout ratio changed from 5 to 25% (by parameter dropout.mid control, from –1.5 to 0.5, and dropout.shape = –1, de.facScale = 0.2). In the imbalanced experiment, we set the number of clusters to five and the total number of cells to 2,500. The cell number in each cluster presents a proportional series, where the proportional coefficient ranges from 0.6 to 1. A smaller proportional coefficient implies that rare cell types are more likely to exist. The number of genes in both experiments was set to be 2500. For the reliability of the experimental results, we generated 10 datasets for each parameter setting and calculated the median of ARI and NMI values in 10 datasets for evaluation. From the overall results of the 25 scenarios in the balanced experiment (see Figure 2A, Figure S1A, and Table 1), the mean ARI(NMI) value of D-scDMFK and scDMFK was 0.87(0.85) and 0.81(0.80), while ZINB, mask MSE, weight MSE and scDeepCluster are 0.79(0.80), 0.39(0.45), 0.76(0.78), and 0.25(0.30), respectively. As for the two other traditional statistical methods, the corresponding values of CIDR and SIMLR were 0.19(0.24) and 0.19(0.34). We could see that D-scDMFK and scDMFK achieved the best performance in both ARI or NMI. Models based on deep-learning and fuzzy k-means algorithm are significantly superior to traditional statistical methods customized for scRNA-seq data. However, another deep learning-based method, scDeepCluseter, showed no obvious advantage. Moreover, based on a fixed dropout ratio, as the number of clusters increased, our two models exhibited better performance in larger cluster number (see Figure 3A and Figure S2A). Conversely, when the cluster number is fixed, then, as dropout ratio increases, the ARI and NMI value of each methods decreases distinctly, but D-scDMFK and scDMFK always came out as the best of three (see Figure 2C and Figure S1C). For the imbalanced experiment (see Figures 2B, 3B, Figures S1B, S2B, and Table 1), the ARI(NMI) values of 15 scenarios for D-scDMFK and scDMFK were 0.86(0.87) and 0.89(0.84). Only weight MSE (ARI 0.87, NMI 0.85) and ZINB (ARI 0.81, NMI 0.87) can match these values, but other methods fall below 0.6. By fixing the dropout rates, as the proportional coefficient decreases, the values of ARI and NMI show a decreasing trend (see Figure 3C and Figure S2C). Despite the existence of rare categories in a small proportional coefficient situation, compared to other methods, D-scDMFK and scDMFK can better address this problem and achieve the best clustering performance. In principle, negative binomial (or adding zero-inflated) distribution is more suitable for fitting Splatter simulation data than multinomial distribution since it is a mixture of poisson distribution with gamma mixing weights. However, our results rebut that idea inasmuch as multinomial modeling is not inferior to the commonly used ZINB model, especially in large cluster number and high dropout rate situations.

**Table 1:** Average performance of eight methods in two groups of simulation datasets and 10 real datasets.

	**Simulation and real data**					
	**Splat balance**		**Splat imbalance**		**Real dataset**	
	**ARI**	**NMI**	**ARI**	**NMI**	**ARI**	**NMI**
CIDR	0.19	0.24	0.35	0.37	0.41	0.50
SIMLR	0.19	0.34	0.20	0.34	0.55	0.67
scDeepCluster	0.25	0.30	0.46	0.54	0.49	0.69
mask MSE	0.39	0.45	0.57	0.58	0.71	0.69
weight MSE	0.76	0.78	0.86	0.85	0.71	0.69
ZINB	0.79	0.80	0.81	**0.87**	0.76	0.77
D-scDMFK	**0.87**	**0.85**	0.86	**0.87**	0.79	0.80
scDMFK	0.81	0.80	**0.89**	0.84	**0.84**	**0.82**

*The bold values represent the maximum value of the corresponding column*.

### 3.3. Analysis of 10 Real Datasets

**Figure 4:**
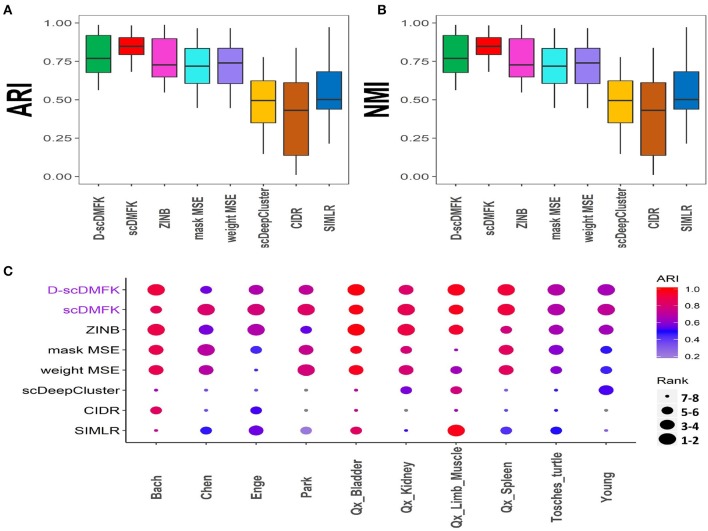
Real data analysis. **(A,B)** Boxplots of ARI and NMI values in 10 real datasets, respectively. **(C)** Dotplot of 10 real datasets. Every point in x-axis stands for a dataset and in y-axis a method. The scatter reflects the corresponding performance of a method in a dataset where the color stands for its ARI value, and the size stands for its ranking according to ARI value among the eight methods. The blue scatter implies that its ARI value is less than 0.2.

To validate the effectiveness of our model on real data, we benchmarked it against other algorithms on 10 published single-cell UMI count datasets from various organs, such as the brain, kidney, pancreas, and so on. The scale of cell number ranges from thousands to tens of thousands, and the proportion of zero counts is above 85%. We used the cell types given by authors as our referenced gold standard labels, which have been verified experimentally. The detailed information for each dataset can be found in Table S1. Considering the influence of random number on the experimental results, we ran each dataset 10 times under different random numbers, and we then took the median value of these ten results as our comparison values (see Figures 4A,B). The average ARI(NMI) value of the 10 datasets taken together (see Table 1) reveals that our models scDMFK and D-scDMFK ranked as the top two with specific values of 0.84(0.82) and 0.79(0.80), respectively, higher than ZINB(ARI 0.76, NMI 0.77), mask MSE(ARI 0.71, NMI 0.69), weight MSE(ARI 0.71, NMI 0.69), scDeepCluster(ARI 0.49, NMI 0.69), and SIMLR(ARI 0.55, NMI 0.67). CIDR performed unsatisfactorily since none of its ARI or NMI values exceeded 0.6. Moreover, our methods ranked in the top three in more than two-thirds of the data and never fell into the last three (see Figure 4C and Figure S3A). A challenging task was the performance of clustering on the “Chen” dataset with 46 cell types, implying many rare cell types. However, scDMFK could achieve ARI 0.82 on it, while other clustering algorithms only gave less than 0.7, illustrating that scDMFK learns a more clustering-friendly low-dimensional representation. Besides, scDMFK also performed well on other datasets with large cluster number, such as “Park”(16 cell types) and “Young”(11 cell types).

**Figure 5:**
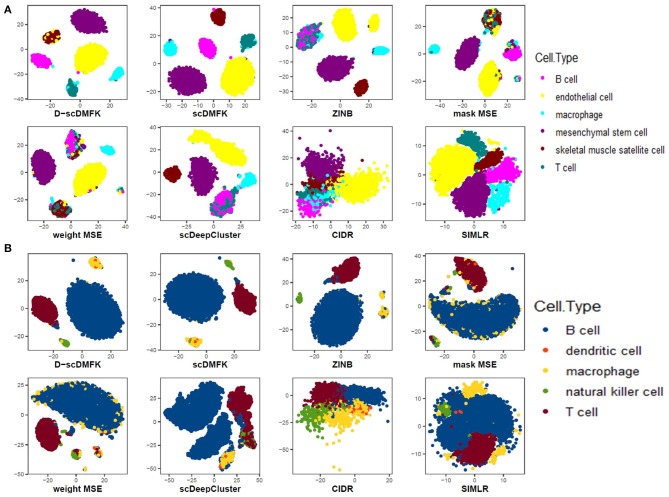
Data visualization in 2D plane. **(A)** Visualization of “Quake_10x_Limb_Muscle” dataset. **(B)** Visualization of “Quake_10x_Spleen” dataset.

We also drew the 2D visualization plots of three 10x genomics datasets, including “Qx_Limb_Muscle,” “Qx_Spleen,” and “Qx_Kidney,” using the t-SNE (Maaten and Hinton, 2008). For deep learning-based methods, we first took their low-dimensional latent space representation and then used t-SNE to reduce it to two dimensions for visualization. For CIDR and SIMLR, we utilized the default visualization procedures in their R packages. Figures 5A,B show the results of “Qx_Limb_Muscle” and “Qx_Spleen” datasets, respectively. For the “Qx_Limb_Muscle” dataset, we can see that D-scDMFK and scDMFK separated those six cell types clearly, while ZINB and mask MSE divided the endothelial cell type into two parts. Besides, ZINB and scDeepCluster could not distinguish B cells and T cells very well, and two MSE-based models encountered a similar situation. CIDR gave the worst visualization since it mixed up the whole cells entirely. For the “Qx_Spleen” dataset, we can see that only D-scDMFK and scDMFK aggregated the B cell and macrophage cell types successfully, while other methods split one or both of them into multiple parts. Another representative example of the “Qx_Kidney” dataset can be found in Figure S3B. Overall, our two models D-scDMFK and scDMFK outperformed other methods in 2D visualization analysis.

### 3.4. Robustness and Scalability

**Figure 6:**
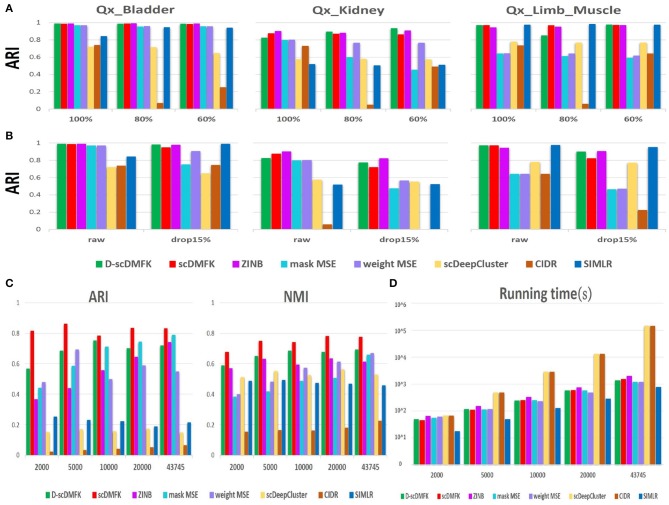
Robustness and scalability experiments of real dataset. **(A)** Downsampling experiments: histogram of ARI values under different sample size of three datasets. **(B)** Dropout experiments: histogram of ARI values for raw data and disturbance data with 15% artificial dropout. **(C)** Scalability experiments. ARI and NMI values in different scale of "Park" data, respectively. **(D)** Scalability experiments. Time consumption in different scale of “Park” data, respectively.

Stability and robustness are essential indicators for evaluating the quality of a clustering algorithm because some algorithms are very sensitive to data disturbances. In this regard, we selected three datasets(“Qx_Bladder,” “Qx_Kidney,” and “Qx_LimbMuscle”) by 10x Genomics sequencing and designed two groups of noise experiments, one to sample a portion of the cells for clustering and another to randomly add zero elements. For the former, we downsampled 60 and 80% cells apart from the total dataset and then ran each clustering algorithm again. For the latter, we randomly masked some non-zero counts into zeros with probability 0.15. We repeated these procedures 10 times and then calculated the median ARI and NMI value to compare with the undisturbed original dataset. In downsampling experiments (see Figure 6A and Figure S4A), scDMFK and D-scDMFK did not show any change in performance, and they were still superior to others. However, the ARI value of CIDR decreased significantly as the downsample proportion increased in the “Qx_LimbMuscle” dataset. In dropout experiments (see Figure 6B and Figure S4B), although almost all methods performed a little worse than noise-free scenarios, scDMFK, D-scDMFK, and ZINB were still the three best models. In general, our proposed models are robust to data disturbances.

With the rapid development of single-cell sequencing technology, the scale of single-cell RNA sequencing data is getting larger and larger, challenging the scalability of clustering algorithms. We therefore chose a mouse kidney dataset, “Park” (Park et al., 2018), as our benchmark dataset, which has 43,745 cells and 16 cell types. We selected 2,000, 5,000, 10,000, 20,000, and total cells, in turn, for clustering analysis, and calculated the ARI and NMI values while recording the running time of each algorithm. No matter the number of cells, we could see that scDMFK always had the highest ARI and NMI value (see Figure 6C). From the point of view of time consumption (see Figure 6D), SIMLR took the least time but got unsatisfactory ARI and NMI values owing to its approximation on large-scale dataset. Our proposed models and MSE-based models also accomplished the whole clustering experiments in the ideal time frame, while ZINB consumed more time since it needs to estimate more parameters and solve more complex likelihood function. CIDR and scDeepCluster are time-consuming, and the latter mainly so due to the absence of preselecting a portion of the genes. Overall, D-scDMFK and scDMFK possess excellent scalability with satisfactory clustering accuracy in large-scale scRNA-seq data analysis.

### 3.5. Disturbance Analysis of Cluster Number

**Figure 7:**
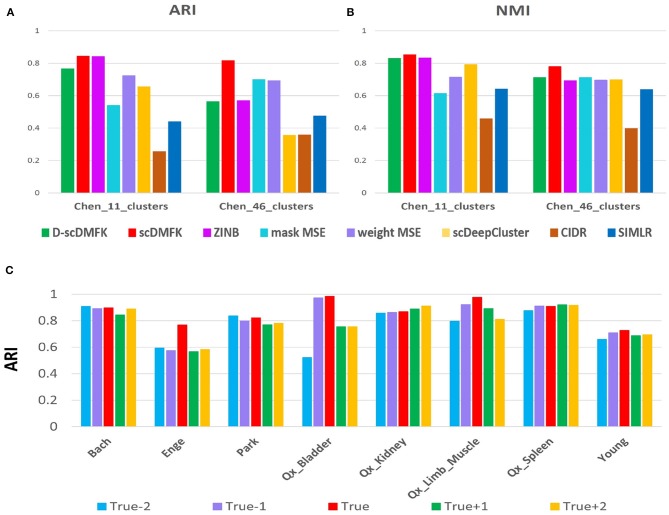
Robustness experiments for changing cluster number. **(A,B)** Comparison of ARI and NMI values in “Chen” dataset with coarse and fine divisions. **(C)** Change of ARI values with the disturbed cluster number in eight real data sets.

In fact, the number of clusters for real datasets can be changed according to the demand of data providers. They can merge some small subclusters into large ones and can also subdivide small ones from some large clusters. In previous experiments, it is worth noting that we used the fine division (46 cell types) provided by the author on the “Chen” dataset. We also performed clustering analysis on its coarse division (11 cell types) given by the author (see Figures 7A,B). Except for mask MSE, CIDR, and SIMLR, other methods have improved the clustering performance when using coarse division as the reference gold standard. In addition, scDMFK performs best in both fine and coarse divisions, indicating that scDMFK has favorable stability when the task involves various fine-grain divisions. Estimating the number of clusters has always been an open problem for statisticians and machine learning researchers. In real data analysis, it is always difficult to obtain a true cluster number in advance. For some datasets, the estimated cluster number by applying gap statistic (Tibshirani et al., 2001) in the learned latent space can be the same as the true cluster number (see Figure S5), while it is overestimated or underestimated in most cases. Therefore, instead of setting a unique number of clusters, we can implement experiments with different cluster numbers for each real dataset. Specially, assuming the true cluster number is *K*, we can utilize scDMFK to perform clustering in referenced cluster number from {*K* − 2, *K* − 1, *K, K* + 1, *K* + 2}. From the ARI and NMI results in Figure 7C and Figure S6, we can see that slight perturbation of cluster number hardly affects the clustering results of scDMFK for those datasets with large true cluster number, such as “Bach,” “Park,” and “Young,” However, for some datasets with a small true cluster number, such as “Qx_Bladder” and “Enge,” changing the referenced cluster number has a more significant effect on the clustering performance, which is reasonable assuming the dataset has only two or three clusters, which will seriously damage its structure. In addition, on some datasets, such as “Qx_Kidney” and “Qx_Spleen,” it can be seen that the referenced cluster number with the optimal clustering performance is not necessarily the true cluster number provided by their authors. This conclusion is consistent with the previously published article (Duó et al., 2018). In general, scDMFK shows satisfactory stability and robustness for perturbation of cluster number.

### 3.6. Effectiveness of Fuzzy Adaptive k-Means Algorithm

**Figure 8:**
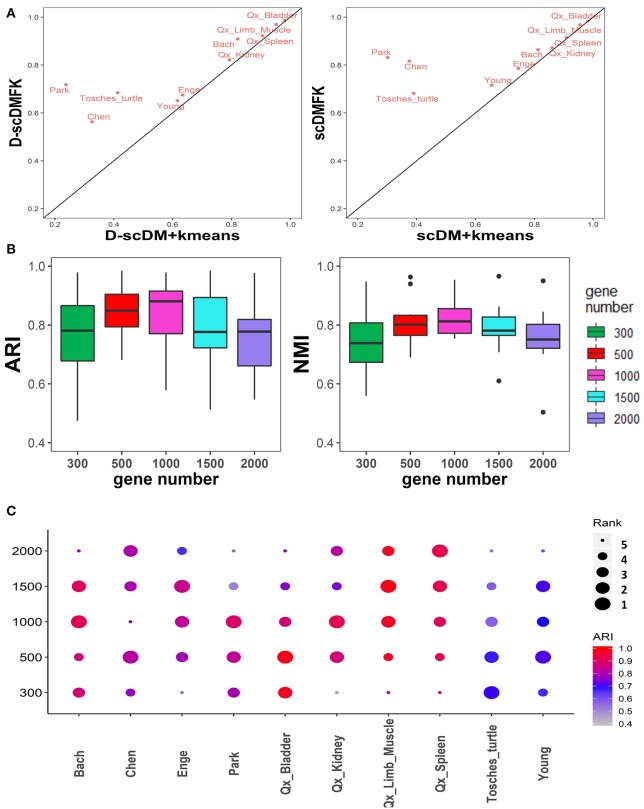
Additional comparison experiments in real datasets. **(A)** Comparison of ARI values between adaptive fuzzy k-means and hard k-means clustering in 10 real datasets. **(B,C)** Boxplot and dotplot of ARI and NMI values in various highly variable genes for 10 real datasets, respectively.

So far, we have not discussed the effect of the fuzzy adaptive k-means clustering on the model because scDMFK, D-scDMFK, ZINB, mask MSE, and weight MSE in the previous experiments were embedded with the fuzzy k-means clustering algorithm simultaneously, which, to a certain extent, explains the superiority of multinomial modeling. Therefore, in this section, we have compared scDMFK and D-scDMFK with the deep multinomial modeling collocating standard k-means clustering algorithm. Specifically, for the latter, we applied only *L*_1_ loss as model optimization objective function and then extracted the well-trained latent representation to perform cell clustering by a standard k-means algorithm. For convenience, we called them scDM+kmeans and D-scDM+kmeans, respectively. Based on the results of 10 real datasets (see Table 2 and Table S2), we found that the average ARI(NMI) values of 10 datasets for D-scDM+kmeans and scDM+kmeans were 0.67(0.73) and 0.70(0.75), respectively, about 10% lower than D-scDMFK(ARI 0.79, NMI 0.80) and scDMFK(ARI 0.84, NMI 0.82). Moreover, D-scDMFK and scDMFK both improve dclustering performance on the basis of D-scDM+kmeans and scDM+kmeans for each dataset (see Figure 8A and Figure S7A), which fully illustrates the necessity of the fuzzy adaptive k-means algorithm.

**Table 2:** Comparison of ARI values among scDMFK, D-scDMFK and scDM+kmeans, D-scDM+kmeans in 10 real datasets.

	**ARI value of real data**			
	**D-scDM+kmeans**	**D-scDMFK**	**scDM+kmeans**	**scDMFK**
Bach	0.82	0.91	0.81	0.87
Chen	0.33	0.56	0.38	0.81
Enge	0.63	0.68	0.75	0.79
Park	0.24	0.72	0.30	0.83
Bladder	0.98	0.99	0.98	0.99
Kidney	0.79	0.82	0.86	0.87
LimbMuscle	0.95	0.97	0.96	0.97
Spleen	0.91	0.92	0.91	0.92
Tosches_turtle	0.41	0.68	0.39	0.68
Young	0.62	0.65	0.65	0.72

*Bladder, Kidney, LimbMuscle, and Spleen refer to Qx_Bladder, Qx_Kidney, Qx_LimbMuscle, and Qx_Spleen, respectively*.

In fuzzy adaptive k-means loss function, σ and λ are two tuning parameters where σ is a distance parameter and controls robustness to outliers, and λ is a weight parameter and controls the distribution of cluster assignment (also controls algorithm convergence speed to some extent). Although we set them both as 1.0 in default, they may not be the optimal parameter setting for some datasets in terms of clustering performance. Thus, we used the popular grid search method in the deep learning model fine-tuning strategy to determine the optimal parameter setting of six small real datasets. Specifically, σ was searched for in the grid of [0.01, 0.1, 1.0, 10, 100], and λ was searched for in the grid of [0.01, 0.1, 1.0]. Table 3 reports the performance comparison between the optimal parameter setting and default scenario. We can easily discover that the ARI and NMI values have been greatly improved under the optimal parameter setting for every real dataset, and saw an average increase of nearly 8%. It is no secret that the model's hyperparameters will affect the clustering effect. Therefore, in the previous method comparison, we used the default parameters of each algorithm, repeatedly ran each method 10 times, and then took their median value to make a more reliable evaluation.

**Table 3:** Optimal parameter settings of scDMFK and D-scDMFK for real datasets.

	**Sigma**	**Lambda**	**ARI(def)**	**ARI**	**NMI(def)**	**NMI**
**scDMFK**						
Enge	1.0	0.01	0.79	0.84	0.75	0.81
Bladder	1.0	0.01	0.98	0.99	0.96	0.98
Kidney	1.0	0.1	0.87	0.96	0.84	0.91
LimbMuscle	0.01	1.0	0.97	0.99	0.94	0.98
Spleen	1.0	1.0	0.92	0.95	0.82	0.87
Young	1.0	1.0	0.72	0.79	0.76	0.81
**D-scDMFK**						
Enge	100	0.01	0.68	0.82	0.73	0.80
Bladder	0.1	0.1	0.99	0.99	0.97	0.98
Kidney	0.01	1.0	0.82	0.98	0.82	0.95
LimbMuscle	0.1	1.0	0.97	0.98	0.94	0.97
Spleen	1.0	0.01	0.92	0.94	0.84	0.87
Young	1.0	1.0	0.65	0.77	0.73	0.80

*The default(abbreviated as def) value of ARI and NMI refers to the median value of 10 repeated tests under default parameter setting. Bladder, Kidney, LimbMuscle, and Spleen refer to Qx_Bladder, Qx_Kidney, Qx_LimbMuscle, and Qx_Spleen, respectively*.

## 4. Discussion and Conclusion

Single-cell sequencing technology allows researchers to explore tissue heterogeneity at the cellular level; of these, transcriptome sequencing is the most commonly used one since it can identify cell types by directly analyzing gene expression data and then discover the key regulatory genes that cause cell heterogeneity. During this process, clustering analysis of cell population is an essential and indispensable procedure. Therefore, in this paper, we have combined deep learning with statistical modeling to construct a novel model that fuses data denoising, data dimensionality reduction, and clustering. For data modeling, we made full use of the generation mechanism of UMI count data and adapt multinomial distribution modeling instead of the widely used negative binomial distribution or its zero-inflation version.

For data dimensionality reduction, we used the neural network (autoencoder) that can efficiently capture the inherent non-linear structure of the data to learn the low-dimensional manifolds where those marker genes are located instead of the linear dimensionality reduction method, PCA, or local structure preservation dimensionality reduction algorithm t-SNE. For cell clustering, we used soft clustering with an adaptive loss function instead of hard clustering. In multiple groups of simulation data generated by the Splatter simulator, including various numbers of cell types, different degrees of sparsity, and different sizes of cell types, our methods were more effective and robust than other deep learning-based and traditional statistic-based scRNA-seq data clustering algorithms. In terms of real data analysis, we selected datasets from different organs, which have diverse population sizes and cell type number. The performance of our methods always ranked in the top three, whether by overall comparison, downsampling comparison, or disturbance factor comparison. On large-scale datasets, our model scDMFK was fast, accurate, and has perfect scalability. It is an effective algorithm for the rapid development of single-cell transcriptome data clustering analysis.

During the analysis of the whole real datasets, we selected 500 highly variable genes as the input of the neural network. We filtered out those genes carrying insufficient information for identifying cell types owing to high noise, and we also hoped to speed up the model calculation process. In fact, we tested the clustering performance of selecting 300, 1,000, 1,500, and 2,000 highly variable genes on real datasets. From the ARI and NMI results in Figure 8B, the results of selecting 500 and 1,000 highly variable genes were superior to other cases on the whole. Specific to each dataset (Figure 8C), selecting 300 or 2,000 highly variable genes would most likely reduce clustering performance to some extent. We recommend that users consider performing clustering experiments on 500 to 1000 highly variable genes.

In addition to CIDR and SIMLR, we also compared scDMFK with other commonly used scRNA-seq data clustering methods, such as Seurat (Satija et al., 2015), SC3 (Kiselev et al., 2017), Raceid (Herman and Grün, 2018), and SOUP (Zhu et al., 2019). Considering that the Seurat method cannot give a specific number of clusters in advance, we ran it several times with its parameter “resolution” changing from 0.5 to 1.5 by 0.1 and took the best ARI and NMI value as its result and recorded the corresponding estimated cluster number. For SC3, we used the approximate version given by the author to test it on large-scale datasets since it was time-consuming on those datasets. In order to reflect the advantage of neural network for dimension reduction, we combined another non-linear dimension reduction algorithm t-SNE with the standard k-means algorithm to perform cell clustering. In addition, considering that scDeepCluster did not perform well in the previous comparative analysis, we suspect that this may be related to its selection of all genes as network inputs; thus here we selected 500 highly variable genes (the same as in scDMFK) as its model input. From the results of Figure S8, scDMFK still performed better than Seurat, SC3, SOUP, Raceid, t-SNE+k-means, and scDeepCluster, with eight datasets ranked first and never dropped out of the top three on all 10 datasets. We found that SC3 performed much worse on large datasets than it does on some small datasets. The unsatisfactory result of Seurat can likely be attributed to its tendency to overestimate cluster number (see Figure S9). The performance of scDeepCluster after selecting highly variable genes has been greatly improved compared to its default version, which fully reflects the importance of selecting informative genes.

From the experimental results, multinomial modeling and fuzzy k-means clustering are indispensable. We replaced the data reconstruction loss with ZINB, mask MSE, and weight MSE for comparison where the overall performance showed that multinomial modeling can better characterize the data structure and facilitate the autoencoder to learn a more appropriate embedded low-dimensional space, and thus be more clustering-friendly. The impact of proper data modeling on clustering analysis is apparent. For example, when mask MSE is utilized as the reconstruction loss, some results of simulation experiments cannot even be compared with CIDR and SIMLR. The fuzzy k-means algorithm is also obviously beneficial to improving the clustering effect. We compared the clustering results between use of standard hard k-means method alone and adaptive fuzzy k-means algorithm where ARI and NMI values of the latter have improved the former by approximately 10%. This is reasonable since the fuzzy k-means algorithm is a probability allocation algorithm and can redress the bias caused by incorrect allocation to a certain extent. On the other hand, the continuity of objective function for clustering is conducive to the optimization process of the overall loss function, thereby avoiding some unnecessary local extreme. The adaptive distance representation can also encourage the model to promote the robustness to outliers and effectively identify rare cell types. Another fascinating phenomenon is that adaptive fuzzy k-means clustering has the potential to be extended to perform trajectory analysis, which can be validated in further research.

Actually, taking advantage of neural network to estimate model parameters is an extraordinarily intriguing method because it relaxes the limitations of the previous bayesian priors (especially conjugate priors) or generalized linear model fitting estimation. DIMM-SC is a such method for single-cell UMI count data clustering based on mixture dirichlet multinomial distribution (Sun et al., 2017). We applied it to perform clustering on six small real datasets and found that it was far less effective than scDMFK where all ARI and NMI values of the six datasets were significantly inferior to scDMFK (see Table 4 and Figure S7B). This fully illustrates the powerful ability in parameter estimation of deep learning technology, which can realize searching the optimal solution in broader solution space.

**Table 4:** Comparison between scDMFK and DIMMSC on six small real datasets.

	**Small real data**			
	**DIMMSC**		**scDMFK**	
	**ARI**	**NMI**	**ARI**	**NMI**
Enge	0.00	0.01	0.79	0.75
Bladder	0.62	0.67	0.98	0.96
Kidney	0.56	0.72	0.87	0.84
LimbMuscle	0.64	0.75	0.97	0.94
Spleen	0.47	0.59	0.92	0.82
Young	0.45	0.59	0.72	0.76

*Bladder, Kidney, LimbMuscle, and Spleen refer to Qx_Bladder, Qx_Kidney, Qx_LimbMuscle, and Qx_Spleen, respectively*.

Nowadays, increasingly mature single-cell sequencing technologies can simultaneously profile genetic, epigenetic, proteomic, and spatial information in individual cells, enabling us to uncover the underlying basis for cellular function and infer causal relationships between various modalities. This provides opportunities and poses challenges for integrative analysis of multiple sources of single-cell data. In the future, we are interested in developing a novel method for single-cell clustering by integrating information from multiple cellular modalities.

## Data Availability Statement

The publicly available real datasets(Bach, Chen, Enge, Park, Qx_Bladder, Qx_Kidney, Qx_LimbMuscle, Qx_Spleen) for this study can be downloaded from the data repository NCBI Gene Expression Omnibus(GSE106273, GSE87544, GSE81547, GSE107585, and GSE109774). The source codes of scDMFK are available at: https://github.com/xuebaliang/scDMFK. For convenience, we also place the whole real datasets in https://github.com/xuebaliang/scDMFK/tree/master/dataset.

## Author Contributions

LC and MD conceived and designed the scDMFK and D-scDMFK models. LC and WW implemented the simulation study and real data set analysis. LC and YZ wrote the whole specific codes. Lastly, LC and MD wrote the whole manuscript. All authors read and approved the final manuscript.

## Conflict of Interest

The authors declare that the research was conducted in the absence of any commercial or financial relationships that could be construed as a potential conflict of interest.
