# Supplementary material for: Single-Cell Transcriptome Data Clustering via Multinomial Modeling and Adaptive Fuzzy K-Means Algorithm
Source: Front Genet. 2020 Apr 17;11:295. doi: 10.3389/fgene.2020.00295 (PMC7180207; doi:10.3389/fgene.2020.00295)
Supplement: Supplementary file 1 [file Data_Sheet_1.pdf]

Single-cell transcriptome data clustering via multinomial modeling and adaptive fuzzy k-means algorithm

1 ADDITIONAL THREE DENOISING MODEL

We substitute zero-inflated negative binomial model, mask MSE and weight MSE for multinomial denoising model, respectively. Particularly, for the former,

$$L_1(\pi_{ij}, \mu_{ij}, \theta_{ij}|X_{ij}) = -\sum_{i=1}^{n}\sum_{j=1}^{m} \log P_{ZINB}(X_{ij}; \pi_{ij}, \mu_{ij}, \theta_{ij}) \tag{S1}$$

where

$$P_{ZINB}(X_{ij}; \pi_{ij}, \mu_{ij}, \theta_{ij}) = \pi_{ij}\delta_0 + (1 - \pi_{ij})P_{NB}(X_{ij}; \mu_{ij}, \theta_{ij}) \tag{S2}$$

$$\delta_0 = I\{X_{ij} = 0\} \tag{S3}$$

$$P_{NB}(X_{ij}; \mu_{ij}, \theta_{ij}) = \frac{\Gamma(X_{ij} + \theta_{ij})}{\Gamma(\theta_{ij})}\left(\frac{\theta_{ij}}{\theta_{ij} + \mu_{ij}}\right)^{\theta_{ij}}\left(\frac{\mu_{ij}}{\theta_{ij} + \mu_{ij}}\right)^{X_{ij}} \tag{S4}$$

For the middle one,

$$L_1(X, \hat{X}) = ||P_X(X - \hat{X})||_F^2 \tag{S5}$$

where operator P_Z forces the loss function only on non-zero entries of X. For the latter,

$$L_1(X, \hat{X}) = \sum_{i=1}^{n}\sum_{j=1}^{m} X_{ij}(X_{ij} - \hat{X}_{ij})^2 \tag{S6}$$

2 ADAPTIVE LOSS FUNCTION

For an arbitrary vector $x \in R^d$, l_1-norm and l_2-norm are defined as $||x||_1 = \sum_{i=1}^{d} |x_i|$ and $||x||_2^2 = \sum_{i=1}^{d} x_i^2$, respectively. As we all know, l_2-norm is sensitive to the large outliers, but has better impact to objective when outliers are small. Conversely, l_1-norm is robust to the large outliers, but sensitive to the small outliers. Similarly, for a matrix $X_{n\times d}$, $l_{2,1}$-norm defined as $||X||_{2,1} = \sum_{i=1}^{n} ||x_i||_2$ is sensitive to the small outliers and robust to the large ones, while the Frobenius-norm represented as $||X||_F^2 = \sum_{i=1}^{n} ||x_i||_2^2$ is sensitive to the large outliers. Besides, $l_{2,1}$-norm is non-smooth, while the Frobenius-norm based optimization problems are easy to solve. To exploit both their advantages, (Nie et al., 2013) proposed a robust loss function namely the adaptive loss function which is defined as

$$||X||_\sigma = \sum_{i=1}^{n} \frac{(1 + \sigma)||x_i||_2^2}{||x_i||_2 + \sigma} \tag{S7}$$

where σ is a tradeoff parameter that controls robustness to various type outliers. We can see that the adaptive loss function interpolates between $l_{2,1}$-norm and Frobenius-norm. And the mathematical properties of $||X_\sigma||$ can be summarized as follows:
1. $||X||_\sigma$ is twice differential, convex and non-negative so that it is suitable as a loss function.
2. When $\forall i$, $||x_i|| \ll \sigma$, then $||X||_\sigma \to \frac{1+\sigma}{\sigma}||X||_F^2$.
3. When $\forall i$, $||x_i|| \gg \sigma$, then $||X||_\sigma \to (1+\sigma)||X||_{2,1}$.
4. When $\sigma \to 0$, then $||X||_\sigma \to ||X||_{2,1}$.
5. When $\sigma \to \infty$, then $||X||_\sigma \to ||X||_F^2$.
Moreover, we also test the performance of $l_{2,1}$-norm and Frobenius-norm on ten real datasets which correspond to $\sigma = 0$ and $\sigma = \infty$, respectively. From the results in Figure S10, we can see that for those datasets with more clusters, such as "Chen", "Park" and "Tosches_turtle", $l_{2,1}$-norm($\sigma = 0$) can result in better clustering performance than Frobenius-norm($\sigma = \infty$).

3 SUPPLEMENTARY TABLES AND FIGURES

3.1 Tables

In the real data analysis section, we select 10 real datasets that have made the purified cell types available to public. We summarize their basic information and source into following table.

Table S1. The information and source for 10 real datasets from different organs. The first column represents the dataset name. Bladder, Kidney, LimbMuscle and Spleen refer to Qx_Bladder, Qx_Kidney, Qx_LimbMuscle and Qx_Spleen, respectively.

	real data information				
	organ	cell type num	cell num	zero percent	reference
Bach	Gammary Gland	8	23184	88.04%	(Bach et al., 2017)
Chen	Brain	46	12089	93.74%	(Chen et al., 2017)
Enge	Pancreas	6	2282	86.05%	(Enge et al., 2017)
Park	Kidney	16	43745	93.60%	(Park et al., 2018)
Bladder	Bladder	4	2500	86.94%	(Schaum et al., 2018)
Kidney	Kidney	8	2781	90.84%	(Schaum et al., 2018)
LimbMuscle	Limb Muscle	6	3909	93.57%	(Schaum et al., 2018)
Spleen	Spleen	5	9552	94.34%	(Schaum et al., 2018)
Tosches_turtle	Brain	15	18664	90.83%	(Tosches et al., 2018)
Young	Kidney	11	5685	94.70%	(Young et al., 2018)

Table S2. Comparison of NMI values among scDMFK, D-scDMFK and scDM+kmeans, D-scDM+kmeans in ten real datasets. Bladder,Kidney,LimbMuscle and Spleen refer to Qx_Bladder, Qx_Kidney, Qx_LimbMuscle and Qx_Spleen respectively.

	NMI value of real data			
	D-scDM+kmeans	D-scDMFK	scDM+kmeans	scDMFK
Bach	0.78	0.86	0.77	0.83
Chen	0.61	0.71	0.62	0.78
Enge	0.71	0.73	0.73	0.76
Park	0.45	0.69	0.49	0.78
Bladder	0.96	0.97	0.96	0.97
Kidney	0.80	0.82	0.82	0.84
LimbMuscle	0.92	0.94	0.92	0.94
Spleen	0.78	0.85	0.80	0.83
Tosches_turtle	0.63	0.68	0.63	0.69
Young	0.71	0.74	0.72	0.76

3.2 Figures

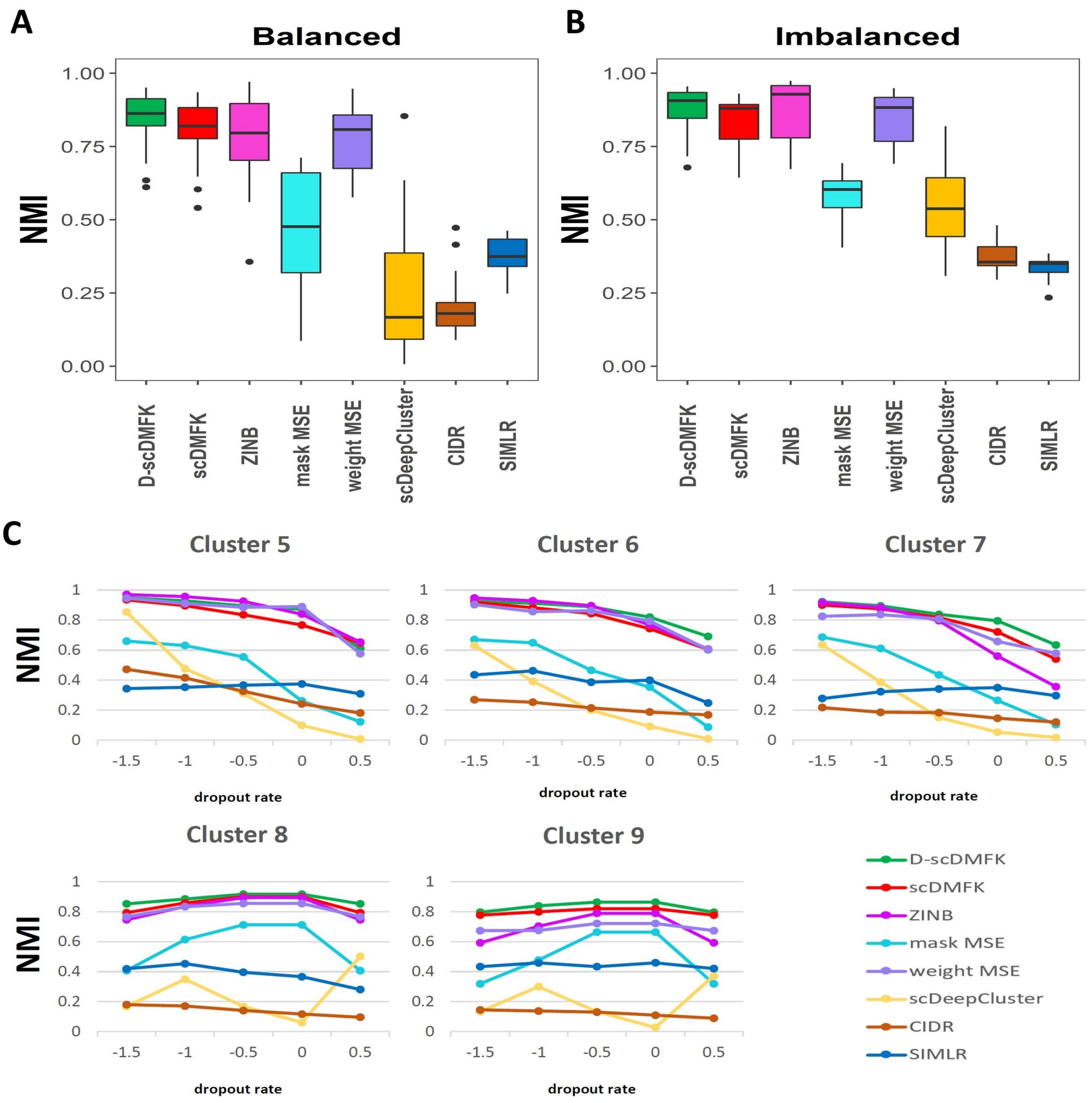

Figure S1. Simulation analysis. (A and B)Boxplots of NMI values in Splatter balanced and imbalanced simulation, respectively. (C)Change of NMI values with the increasing dropout rate in Splatter balanced experiment.

REFERENCES

Bach, K., Pensa, S., Grzelak, M., Hadfield, J., Adams, D. J., Marioni, J. C., et al. (2017). Differentiation dynamics of mammary epithelial cells revealed by single-cell RNA sequencing. Nature Commun. 8, 2128. doi: 10.1038/s41467-017-02001-5.

Chen, R., Wu, X., Jiang, L., Zhang, Y. (2017). Single-cell RNA-seq reveals hypothalamic cell diversity. Cell Rep. 18, 3227-3241. doi: 10.1016/j.celrep.2017.03.004.

Enge, M., Arda, H. E., Mignardi, M., Beausang, J., Bottino, R., Kim, S. K., et al. (2017). Single-cell analysis of human pancreas reveals transcriptional signatures of aging and somatic mutation patterns. Cell. 171, 321-330. doi:10.1016/j.cell.2017.09.004

Nie, F., Wang, H., Huang, H., Ding, C. (2013). Adaptive loss minimization for semi-supervised elastic embedding. IJCAI.

Park, J., Shrestha, R., Qiu, C., Kondo, A., Huang, S., Werth, M., et al. (2018). Single-cell transcriptomics of the mouse kidney reveals potential cellular targets of kidney disease. Science. 360, 758-763. doi:10.1126/science.aar2131.

Schaum, N., Karkanias, J., Neff, N. F., May, A. P., Quake, S. R., Wyss-Coray, T., et al. (2018). Single-cell transcriptomics of 20 mouse organs creates a Tabula Muris: The Tabula Muris Consortium. Nature. 562, 367. doi: 10.1038/s41586-018-0590-4.

Tosches, M. A., Yamawaki, T. M., Naumann, R. K., Jacobi, A. A., Tushev, G., Laurent, G. (2018). Evolution of pallium, hippocampus, and cortical cell types revealed by single-cell transcriptomics in reptiles. Science. 360, 881-888. doi: 10.1126/science.aar4237.

Young, M. D., Mitchell, T. J., Braga, F. A. V., Tran, M. G., Stewart, B. J., Ferdinand, J. R., et al.(2018). Single-cell transcriptomes from human kidneys reveal the cellular identity of renal tumors. Science. 361, 594-599. doi: 10.1126/science.aat1699.

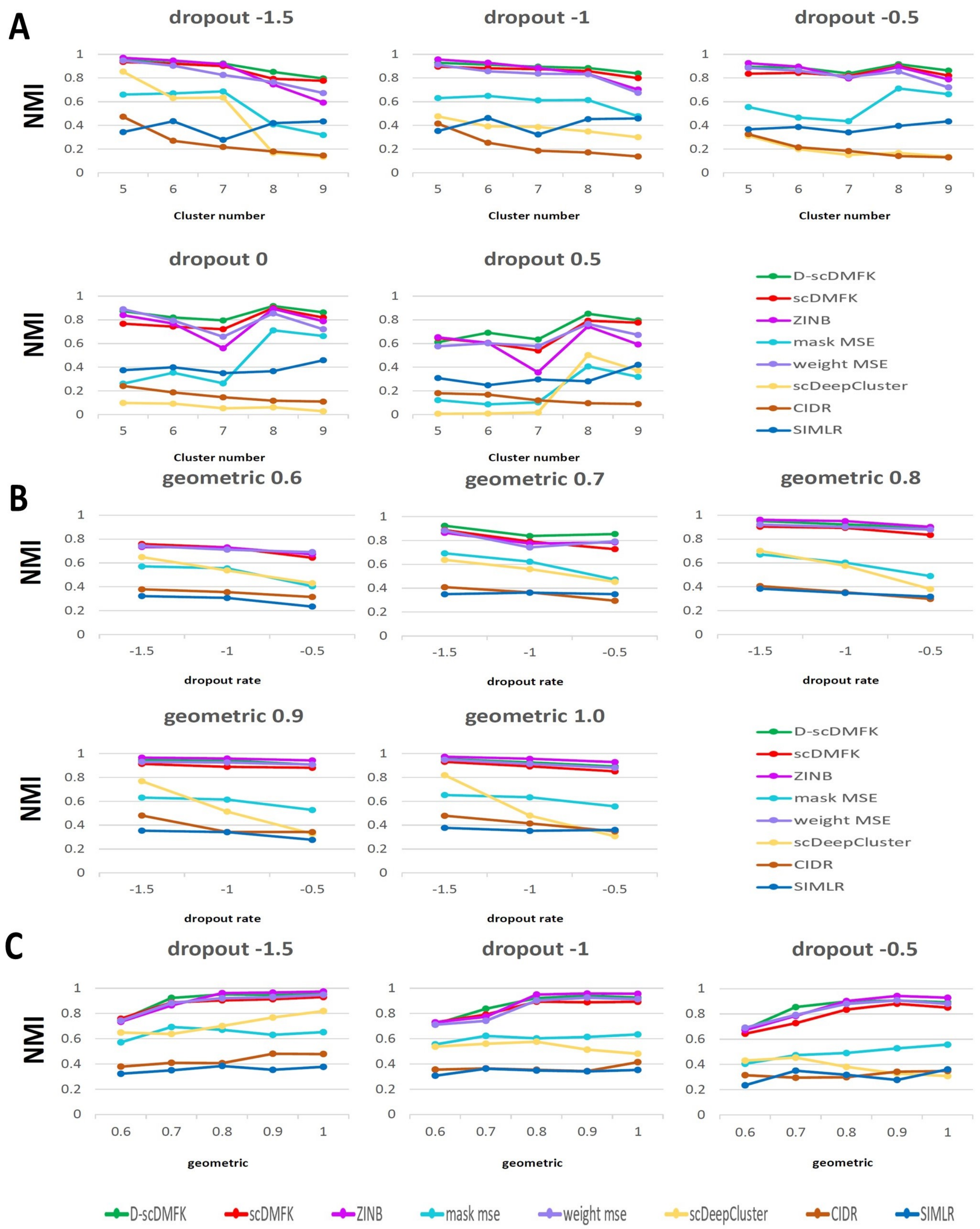

Figure S2. Simulation analysis. (A)Change of NMI values with the increasing cluster number in Splatter balanced experiment. (B and C)Change of NMI values with the increasing dropout rate and geometric in Splatter imbalanced experiment.

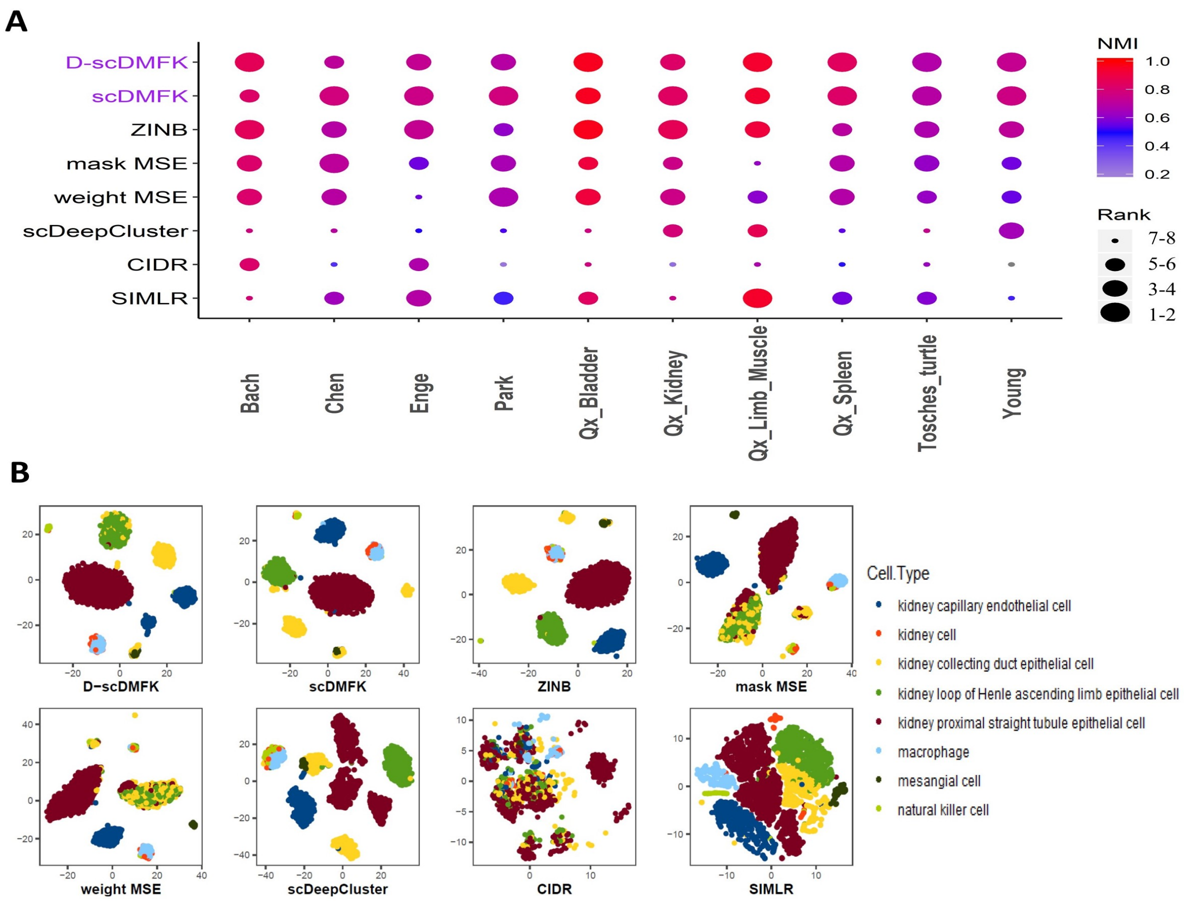

Figure S3. Real data analysis. (A)Dotplot of ten real datasets. Every point in x-axis stands for a dataset and in y-axis a method. The scatter reflects the corresponding performance of a method in a dataset where the color stands for its NMI value, and the size stands for its ranking according to NMI value among the eight methods. The blue scatter implies that its NMI value is less than 0.2. (B) Visualization of "Quake_10x_Kidney" dataset.

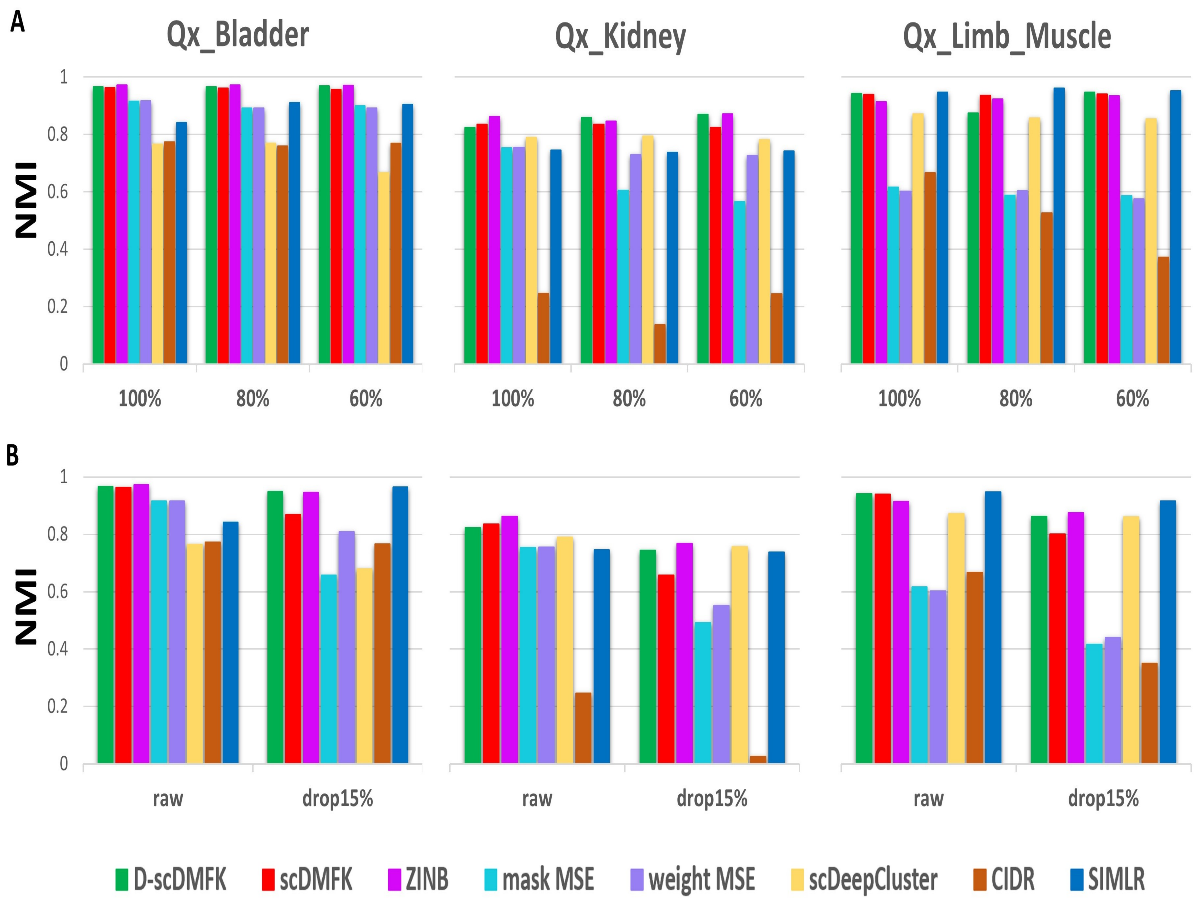

Figure S4. Robustness and scalability experiments of real dataset. (A) Downsampling experiments: histogram of NMI values under different sample size of three datasets.(B) Dropout experiments: histogram of NMI values for raw data and disturbance data with 15% artificial dropout.

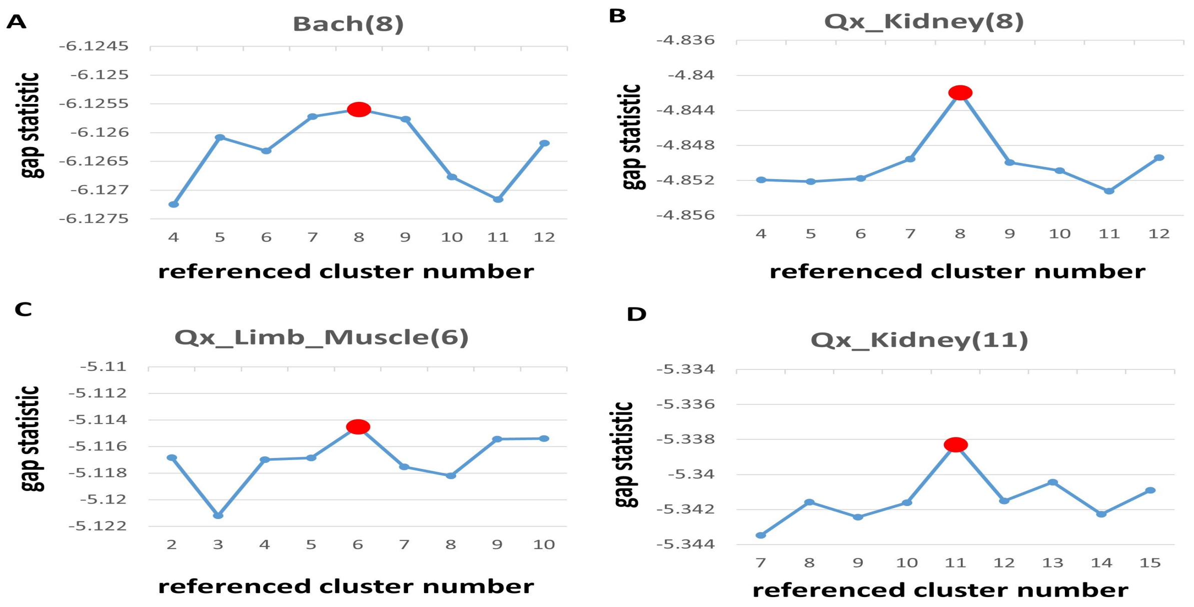

Figure S5. Cluster number estimation experiments in real datasets using gap statistic. (A, B, C and D)Change of gap statistic values(y-axis) with the referenced cluster number(x-axis) in four real datasets. The number in parentheses represents the true cluster number for the corresponding dataset. The red dot corresponds to the optimal cluster number estimated by gap statistic, which is consistent with the true one.

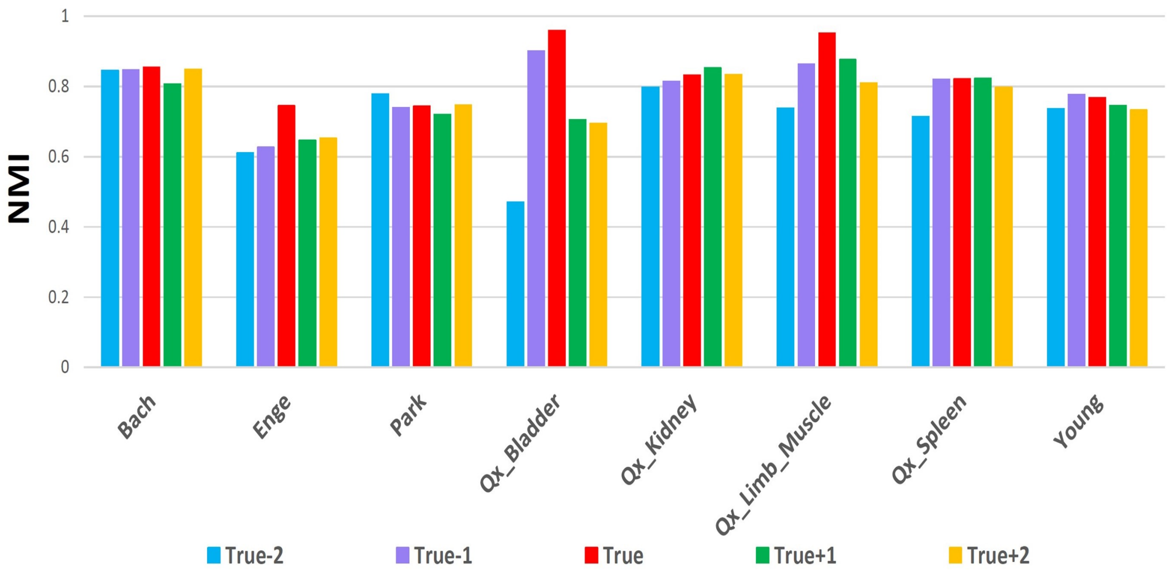

Figure S6. Robustness experiments for changing cluster number. Change of NMI values with the disturbed cluster number in eight real data sets.

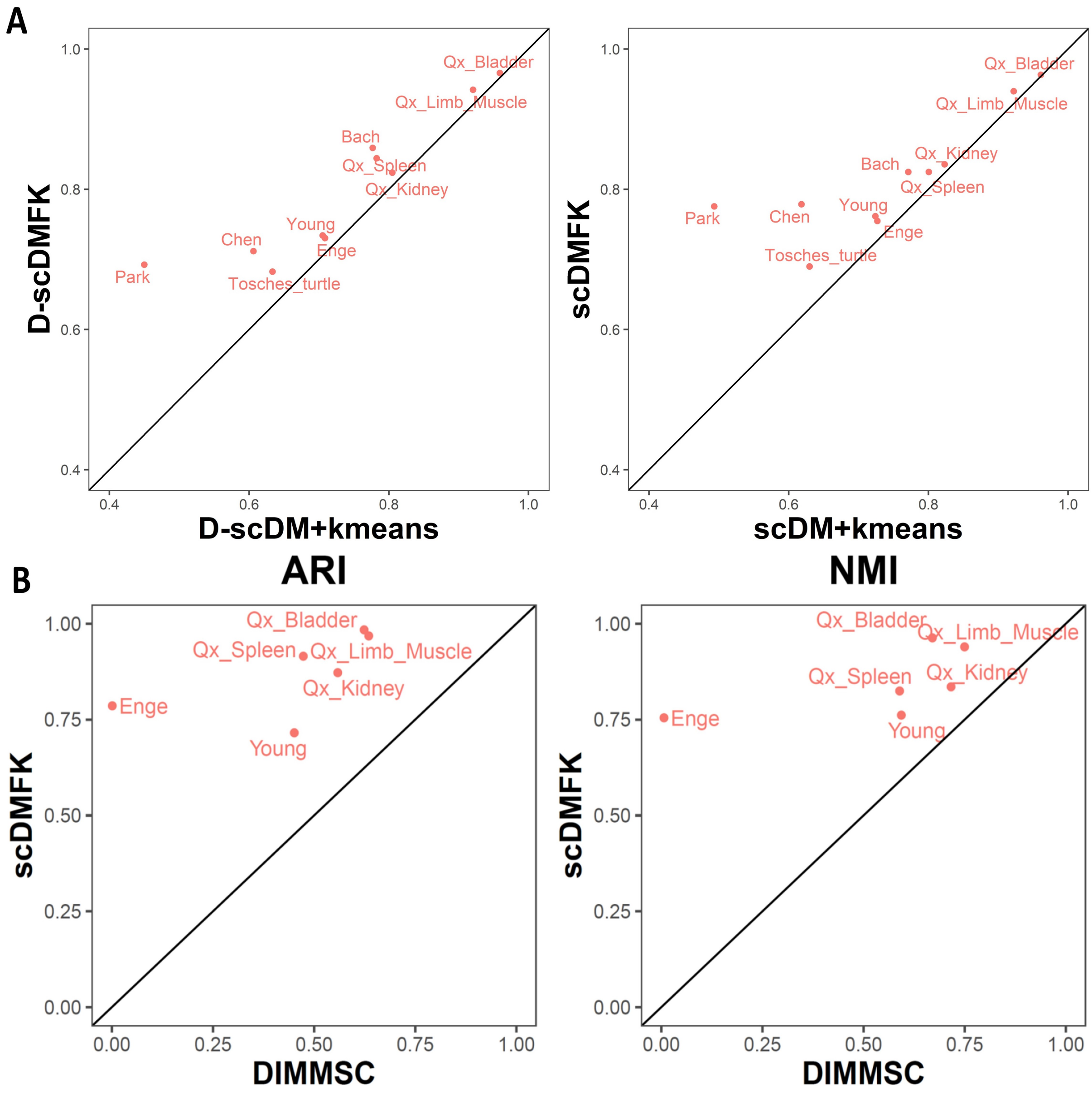

Figure S7. Additional comparison experiments in real datasets. (A)Comparison of NMI values between adaptive fuzzy k-means and hard k-means clustering in ten real datasets. (B)Comparison of ARI and NMI values between scDMFK and DIMMSC in six real datasets, respectively.

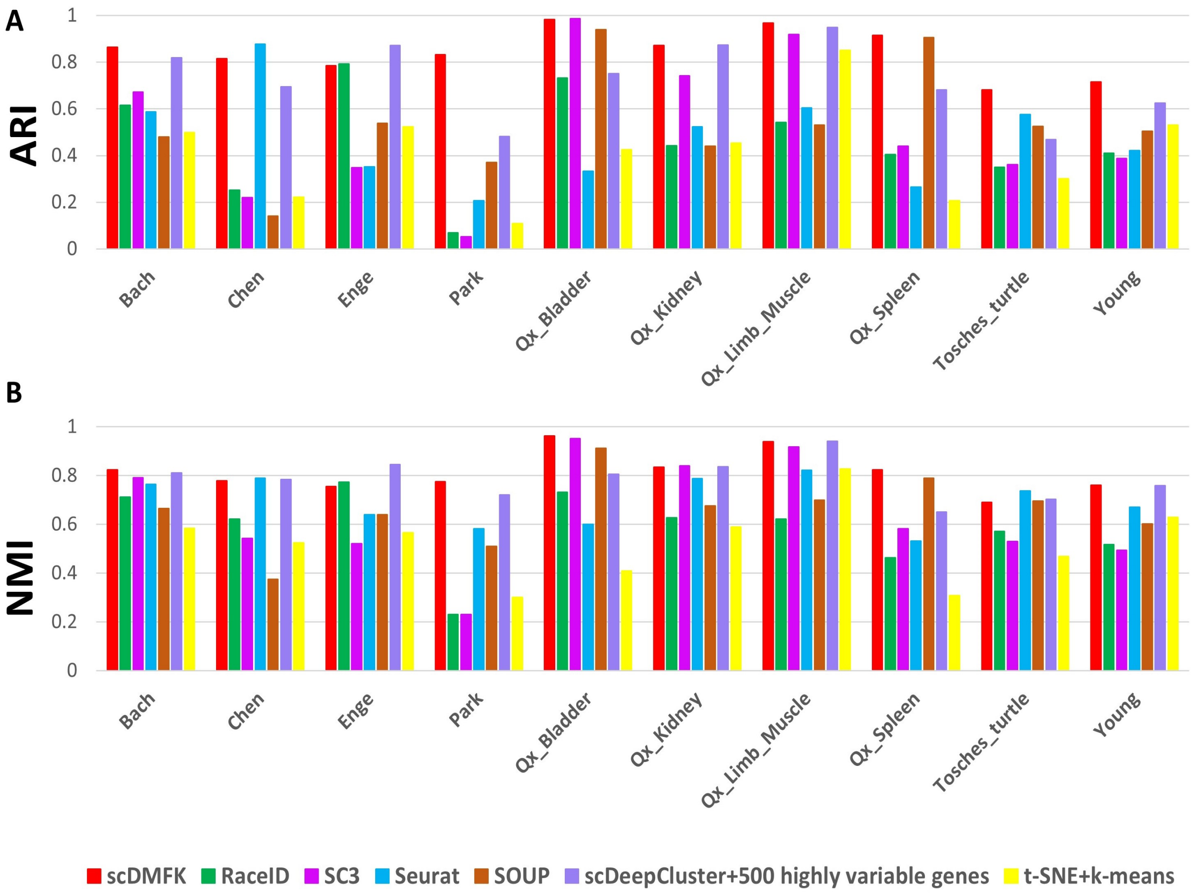

Figure S8. Additional comparison experiments in real datasets. (A and B)Comparison of ARI and NMI values between scDMFK and other scRNA-seq data clustering methods in ten real datasets.

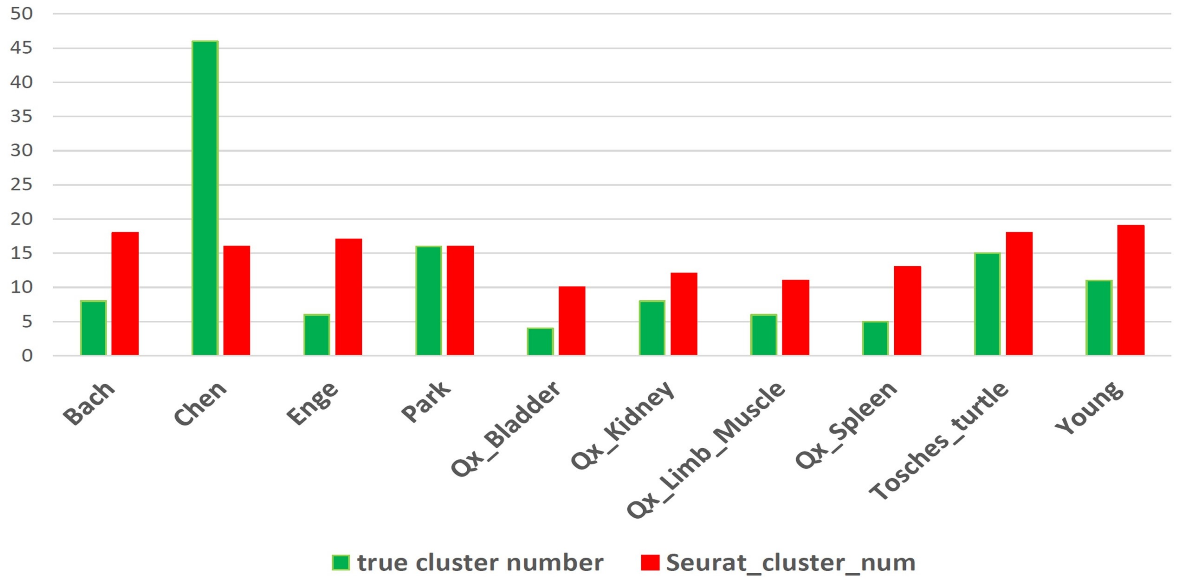

Figure S9. Additional comparison experiments in real datasets. Comparison of true cluster numbers and estimated ones by Seurat in ten real datasets.

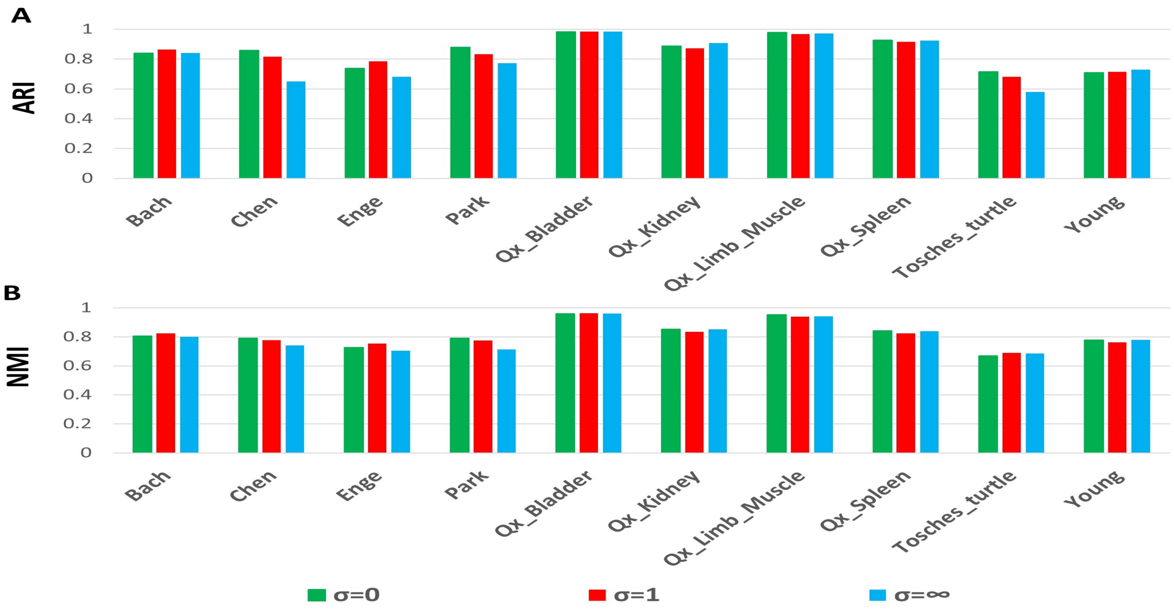

Figure S10. Additional comparison experiments in real datasets. (A and B)Change of ARI and NMI values with three σ value situations in ten real datasets.
